# Local late gadolinium enhancement features to identify the electrophysiological substrate of post-infarction ventricular tachycardia: a machine learning approach

**DOI:** 10.1186/1532-429X-17-S1-P234

**Published:** 2015-02-03

**Authors:** Rocio Cabrera Lozoya, Jan Margeta, Loic Le Folgoc, Yuki Komatsu, Benjamin Berte, Jatin S Relan, Hubert Cochet, Michel Haïssaguerre, Pierre Jaïs, Nicholas Ayache, Maxime Sermesant

**Affiliations:** Asclepios team, Inria, Sophia Antipolis, France; Institut LYRIC, Hôpital Cardiologique du Haut-L’évêque, l’Université Victor Segalen Bordeaux II, Bordeaux, France

## Background

Most ventricular tachycardias occur on structurally diseased hearts with fibrotic scar, where bundles of surviving tissue promote electrical circuit re-entry. These bundles can be identified on invasive electrophysiological (EP) mapping as local abnormal ventricular activities (LAVA) during sinus rhythm. Although the elimination of LAVAs by radiofrequency ablation was shown to be an efficient therapeutic option, their identification requires is a lengthy and invasive process. Late gadolinium enhancement (LGE) magnetic resonance imaging enables a non-invasive 3D assessment of scar topology and heterogeneity with millimetric spatial resolution. The aim of this work is to identify imaging features associated with LAVA, features that may subsequently be used to target ablation or to stratify the risk of arrhythmia.

## Methods

We studied three patients presenting post-infarction ventricular tachycardia and referred for catheter ablation. LGE imaging was performed on a 1.5T scanner using a respiratory navigated and inversion recovery prepared 3D turboFLASH method with high spatial resolution (1.25x1.25x2.5mm). Electroanatomical mapping was performed at high-density during sinus rhythm using a multi-spline catheter and a 3D catheter localization system. Electrograms were categorized as normal or LAVA by an experienced electrophysiologist. Annotated electroanatomical maps were registered to MRI data using an iterative closest point algorithm, with the addition of manual registration when needed. A machine-learning algorithm (random forests) was used to identify local image features (based on texture and intensity) associated with LAVA. A formal integration of the uncertainty related to catheter motion and data registration was implemented, and the impact of this approach on the predictive performance was assessed.

## Results

**Figure 1:**
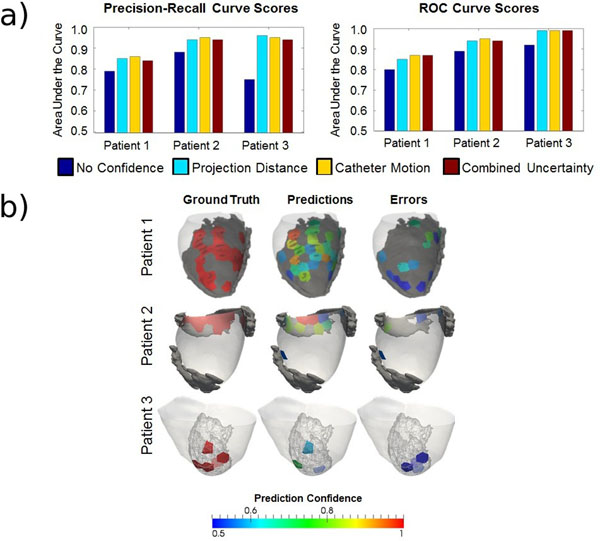
a) Bar plots for classification performance scores using sample uncertainty weights b) [left] LAVA regions from ground truth. [center] LAVA regions from predictions. [right] Prediction errors. (Color coding by classification confidence)

Mean mapping density was 99 points per map. Out of a total of 298 electrograms, 81 had been categorized as abnormal and targeted by ablation. Results of classification with the full feature set and weighted samples, using the mean area under the curve (AUC) for precision-recall (PR) and receiver operating characteristic (ROC) curves, are shown in Figure 1a and Table 1. A visual interpretation of the results is shown in Figure 1b. LAVAs could be predicted from local LGE imaging features with areas under ROC curves ranging from 0.8 to 0.9. The integration of the uncertainty related to catheter motion and data registration improved the accuracy of predictions in all cases.

**Tab1e 1:** Classification performance scores using sample uncertainty weights

Area under the curves	Patient 1		Patient 2		Patient 3	
	**PR**	**ROC**	**PR**	**ROC**	**PR**	**ROC**
**No confidence weighting**	0.79	0.80	0.88	0.89	0.75	0.92
**Project distance weighting**	0.85	0.85	0.94	0.94	0.96	0.99
**Temporal displacement weighting**	0.86	0.87	0.95	0.95	0.95	0.99
**Combined uncertainty weighting**	0.84	0.87	0.94	0.94	0.94	0.99

## Conclusions

The prediction of ventricular tachycardia ablation targets is feasible using locally computed imaging features from high-resolution LGE imaging. The integration of the uncertainty related to catheter motion and data registration improves the predictive performance. A framework is proposed for data analysis and results display.

## Funding

Part of this work was funded by the European Research Council through the ERC Advanced Grant MedYMA 2011-291080.

